# Establishment of a Risk Scoring Model for Perioperative Unex-Plained Shock during Percutaneous Coronary Intervention for the Treatment of Chronic Total Occlusion

**DOI:** 10.31083/j.rcm2310342

**Published:** 2022-10-11

**Authors:** Zichao Cheng, Hongyu Peng, Wen Jian, Yanci Liu, Haiwei Li, Songyuan He, Yingkai Li, Yuchao Zhang, Yuchen Shi, Jinghua Liu

**Affiliations:** ^1^Center for Coronary Artery Disease (CCAD), Beijing Anzhen Hospital, and Beijing Institute of Heart, Lung and Blood Vessel Diseases, Capital Medical University, 100029 Beijing, China

**Keywords:** chronic total occlusion, complication, shock, percutaneous coronary intervention

## Abstract

**Background:**

Several complications can contribute to the risk of shock during the chronic total occlusion (CTO) percutaneous coronary intervention (PCI) procedure. However, some patients that develop shock do not exhibit any apparent complications, and few studies to date have discussed the risk of unexplained perioperative shock in patients undergoing CTO PCI. Accordingly, this study was designed with the goal of defining perioperative risk factors linked to the odds of unexplained shock during CTO PCI.

**Methods:**

In total, this study analyzed data from 924 patients that underwent CTO PCI without any in-hospital complications from January 2016–August 2021. Cardiologists collected data pertaining to patient clinical characteristics, laboratory findings, angiographic findings, and procedural characteristics. Patients were separated into two groups based upon whether or not they experienced perioperative shock. The relationship between specific variables and perioperative shock incidence was assessed via a multivariable stepwise logistic regression approach. A risk-scoring nomogram was then designed for use as a tool to guide patient risk assessment efforts during PCI procedural planning.

**Results:**

Overall, 4.8% of these patients (44/924) experienced unexplained perioperative shock. Independent predictors associated with unexplained shock during CTO PCI included baseline systolic pressure (odds ratio (OR) 0.968, 95% confidence interval (CI): 0.945–0.991), baseline heart rate (OR 1.055, 95% CI: 1.020–1.091), baseline hemoglobin (OR 0.970, 95% CI: 0.947–0.994), procedure duration (OR 1.008, 95% CI: 1.002–1.015), J-CTO score (OR 1.521, 95% CI: 1.021–2.267), and use of a retrograde approach (OR 3.252, 95% CI: 1.426–7.415). The unbiased C-index estimate was 0.859, and this model exhibited excellent calibration.

**Conclusions:**

The risk of unexplained shock is an important consideration for clinicians performing the CTO PCI procedure. These analyses revealed unexplained shock risk to be independently related to lower baseline systolic pressure, higher baseline heart rate, lower baseline hemoglobin, more procedure time, higher J-CTO score, and more use of a retrograde approach.

## 1. Introduction

Several new techniques and pieces of equipment have been developed over the past 10 years to overcome to complexities inherent to the chronic total occlusion (CTO) percutaneous coronary intervention (PCI) procedure, thereby improving operative success rates [1, 2]. When successful, CTO PCI can contribute to the alleviation of patient symptoms and prolonged survival [3, 4]. However, the utility of the CTO PCI procedure is limited by the potential for serious complications that can negatively impact patient prognosis [5, 6]. In severe cases, patients may exhibit cardiac or non-cardiac complications that can contribute to the incidence of hypotension and potentially circulatory shock [7]. Despite these risks, hypotension and shock are often not included in lists of procedure-related complications in published studies [2] and scoring systems. In clinical settings, a subset of patients who experience shock do not exhibit any serious CTO PCI complications, and shock can even occur in a subset of patients who undergo successful CTO PCI treatment, necessitating the prolonged use of vasoactive drugs to maintain appropriate blood pressure. No reports to data have described this form of unexplained perioperative shock associated with the CTO PCI procedure. As such, this retrospective study was designed to survey the incidence of unexplained CTO PCI-related shock and to identify associated risk factors.

## 2. Methods

### 2.1 Patient Population

**Fig. 1.**
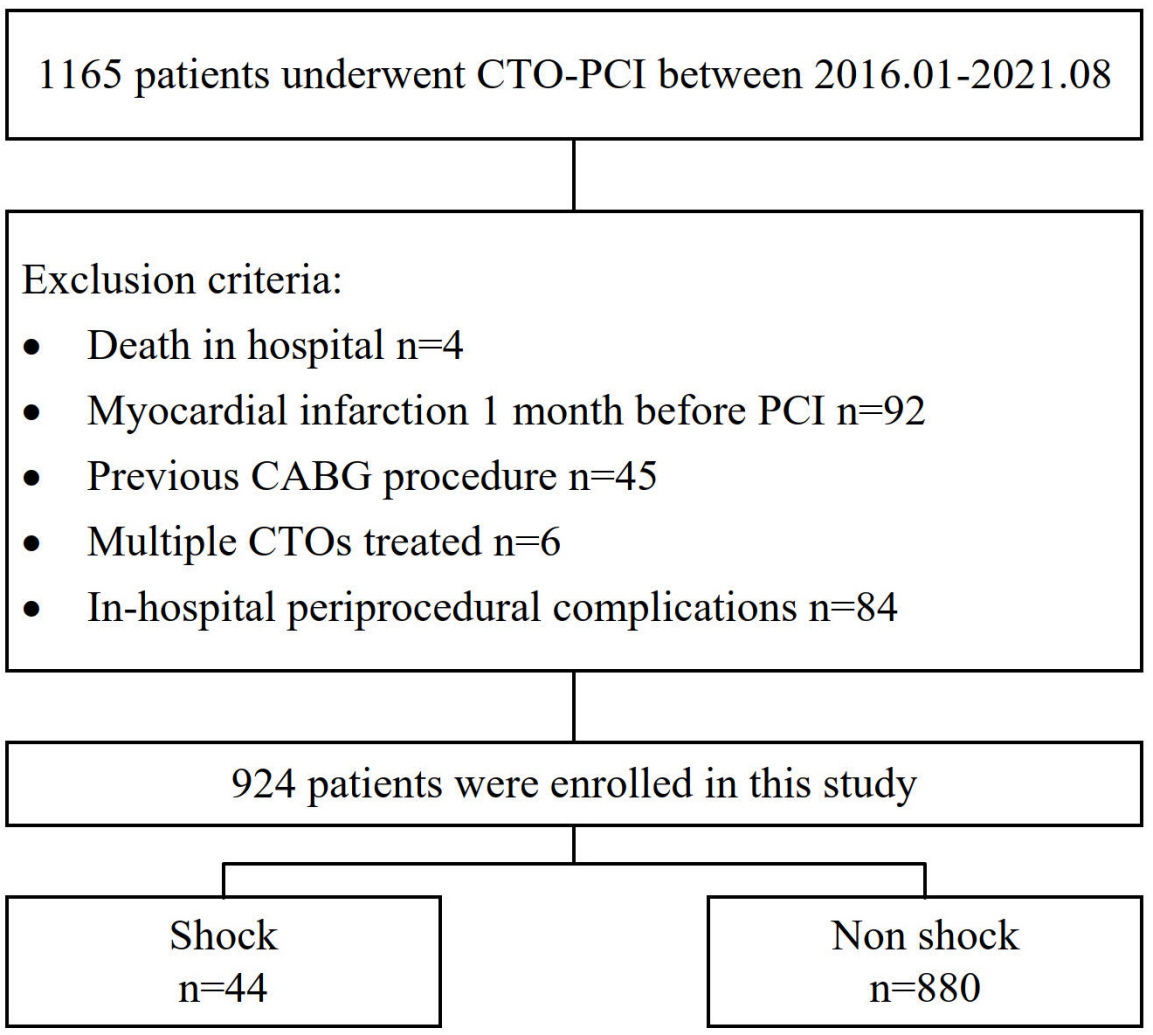
**Flow chart of this study**.

This retrospective analysis incorporated data from 1165 consecutive patients that underwent the CTO PCI procedure in Beijing Anzhen Hospital, Capital Medical University from January 2016 to August 2021. Of these patients, 924 were ultimately included in this study based on defined inclusion/exclusion criteria. These patients were separated into two groups based upon they did or did not develop perioperative shock (n = 44 and n = 880, respectively). The study flowchart is shown in Fig. 1, while in-hospital periprocedural complications are detailed in Table 1. The Institutional Review Board of our center approved this study. All CTO PCI procedures were performed by an experienced team of cardiologists.

**Table 1.** **Rates of in-hospital periprocedural complications**.

Complication	n = 84
No-reflow or slow-flow	n = 2
Arrhythmia requiring treatment	n = 4
Coronary perforation	n = 14
Donor vessel injury	n = 8
Acute thrombosis	n = 2
Vascular access complications	n = 18
Major bleeding*	n = 48
Vascular access complications & bleeding	n = 6

One patient was major bleeding with coronary perforation; one patient was major bleeding with donor vessel injury. *A drop in the hemoglobin of ≥3.0 g/dL or requiring transfusion of ≥3 U of whole blood.

### 2.2 Definitions

Shock was defined as systolic blood pressure (SBP) <90 mmHg for >30 min or the need for supportive intervention to maintain SBP >90 mmHg with evidence of end-organ damage [8]. CTO PCI and related complications were defined based on the 2021 ARC CTO definition [9]. Specifically, CTO was defined as an occlusive coronary lesion with a TIMI (thrombolysis in myocardial infarction) grade of 0 for a minimum of 3 months. Occlusion duration was estimated in-clinic based on patient history of myocardial infarction (MI) in the region of the target vessel, initial angina onset, or comparisons with previous angiograms [9].

CTO PCI technical success was defined by achieving a TIMI grade ≥2 with antegrade flow in all ≥2.5-mm distal branches and with <30% residual stenosis of the target CTO lesion upon procedural completion [9]. Procedural success was defined by both technical success and the absence of any in-hospital major adverse cardiovascular events (MACEs, including MI, clinically necessary target vessel revascularization [TVR], or death) [9]. Major bleeding was defined as a ≥3.0 g/dL decrease in hemoglobin levels or the need for the transfusion of ≥3 U of whole blood, as per CTO-ARC Consensus recommendations [10]. Operative duration was defined as the time from successful puncture to the completion of the final angiographic evaluation.

### 2.3 Statistical Analysis

Categorical variables are reported as percentages and were compared via Fisher’s exact test or Pearson chi-square tests, whereas continuous variables are reported as means ± standard deviation and were compared via Wilcoxon rank-sum tests or *t*-tests. Independent predictors of unexplained shock risk were predicted through univariate and multivariate logistic regression analyses. A nomogram was constructed based on identified predictors, with the calibration and discriminatory ability of this model being assessed using 1000 bootstrap replicates based on the sample size as used for the final model. Data are reported in the form of odds ratios (ORs) with 95% confidence intervals (CIs). All data were analyzed using SPSS 25.0 (IBM Corp., Chicago, IL, USA.) and R Studio (Version: 4.1.2, Boston, MA, USA), with a two-sided *p *< 0.05 as the threshold of significance.

## 3. Results

### 3.1 Baseline Patient Characteristics

The majority of patients included in this study were male and of Asian ethnicity. Of these patients 69.1% had hypertension, 48.9% had undergone prior PCI, and 35.6% were diabetic. The mean ejection fraction for these patients was 60.1% ± 9.1%. Relative to those patients that did not experience shock while undergoing CTO PCI, those patients that did experience shock were more likely to exhibit a lower BMI (*p* = 0.049), a lower baseline SBP (*p* = 0.029), a faster baseline heart rate (*p* = 0.022), to have undergone prior PCI (*p* = 0.045), and to suffer from moderate-to-severe valvular regurgitation (*p* = 0.044). For further details regarding patient characteristics, see Table 2.

**Table 2.** **Baseline clinical characteristics of patients in this study**.

Variable	Overall	Shock		*p* value
	(n = 924)	Yes (n = 44)	No (n = 880)	
Gender (Female)	168 (18.4%)	9 (20.0%)	159 (18.3%)	0.689
Age, y	58.4 ± 10.4	56.8 ± 10.3	58.5 ± 10.4	0.264
BMI, kg/$m^{2}$	26.4 ± 3.5	25.3 ± 4.6	26.5 ± 3.5	0.049
Baseline systolic pressure, mmHg	127.9 ± 16.2	122.8 ± 19.0	128.1 ± 16.0	0.029
Baseline diastolic pressure, mmHg	72.6 ± 10.6	70.8 ± 11.3	72.7 ± 10.6	0.268
Baseline heart rate, /min	71.9 ± 10.8	76.1 ± 12.5	71.7 ± 10.6	0.022
Hypertension	636 (69.1%)	28 (63.6%)	608 (69.1%)	0.446
Diabetes mellitus	326 (35.3%)	11 (25.0%)	315 (35.8%)	0.144
Dyslipidemia	766 (82.9%)	34 (77.3%)	732 (83.2%)	0.310
Prior stroke	40 (4.3%)	0 (0%)	40 (4.5%)	0.148
Atrial fibrillation	10 (1.1%)	2 (4.5%)	8 (0.9%)	0.078
Prior myocardial infarction	246 (26.6%)	15 (34.1%)	231 (26.2%)	0.251
Prior PCI	452 (48.9%)	28 (63.6%)	424 (48.2%)	0.045
Current tobacco use	367 (39.7%)	16 (36.4%)	351 (39.9%)	0.641
**Echocardiography**				
Ejection fraction	60.1 ± 9.1	60.3 ± 7.0	60.1 ± 9.2	0.586
Ejection fraction <40%	29 (3.1%)	0 (0%)	29 (3.3%)	0.392
Ejection fraction <50%	92 (10.0%)	3 (6.8%)	89 (10.1%)	0.609
LVEDD	49.0 ± 5.8	48.2 ± 5.7	49.0 ± 5.8	0.648
LVESD	32.8 ± 6.8	32.8 ± 5.6	32.8 ± 6.9	0.263
Ventricular aneurysm	42 (4.5%)	0 (0%)	42 (4.8%)	0.260
Valvular regurgitation (moderate-severe)	42 (4.5%)	5 (11.4%)	37 (4.2%)	0.044
**Medication**				
Aspirin	924 (100%)	44 (100%)	880 (100%)	-
Clopidogrel	679 (73.9%)	30 (68.2%)	649 (73.7%)	0.414
Ticagrelor	245 (26.5%)	14 (31.8%)	231 (26.3%)	0.414
ACEI/ARB	436 (47.2%)	17 (38.6%)	419 (47.6%)	0.244
β-blocker	599 (64.8%)	33 (75.0%)	566 (63.2%)	0.148
Nitrates	713 (77.2%)	35 (79.5%)	678 (77.0%)	0.700
Calcium channel blocker	274 (29.7%)	12 (27.3%)	262 (29.8%)	0.723
Loop diuretics	108 (11.7%)	5 (11.4%)	103 (11.7%)	0.945
Statin	898 (97.2%)	44 (100%)	854 (97.0%)	0.247
Aldosterone receptor antagonist	58 (6.3%)	4 (9.1%)	54 (6.1%)	0.350
Oral anticoagulants	12 (1.3%)	2 (4.5%)	10 (1.1%)	0.108
Low molecular heparin	123 (13.3%)	7 (15.9%)	116 (13.2%)	0.603

BMI, body mass index; ACEI, angiotensin converting enzyme inhibitors; ARB, angiotensin receptor blocker; LVEDD, left ventricular end diastolic diameter; LVESD, left ventricular end systolic diameter; PCI, percutaneous coronary intervention.

### 3.2 Laboratory Examinations 

Relative to patients that did not experience shock, those that did experience shock exhibited lower baseline RBC levels (4.4 ± 0.5 vs. 4.6 ± 0.5, *p* = 0.021), postoperative RBC levels (3.9 ± 0.6 vs. 4.4 ± 0.5, *p *< 0.001), baseline Hb levels (135.8 ± 14.4 vs. 140.8 ± 14.8, *p* = 0.030), and postoperative Hb levels (120.0 ± 17.6 vs. 133.2 ± 15.7, *p *< 0.001). In the overall patient cohort, the mean decrease in Hb levels (ΔHb) was 8.0 ± 8.7 g/L, while this decrease was greater among patients that experienced shock (15.6 ± 8.6 vs. 7.6 ± 8.5, *p *< 0.001). LDL-C levels were also significantly lower among patients in the shock group (2.0 ± 1.0 vs. 2.2 ± 0.9, *p* = 0.040). For further details regarding patient laboratory findings, see Table 3.

**Table 3.** **Laboratory examinations of patients in this study**.

Variable	Overall	Shock		*p* value
	(n = 924)	Yes (n = 44)	No (n = 880)	
WBC, ${\mathrm{10}}^{9}$/L	6.9 ± 1.9	6.8 ± 2.1	6.9 ± 1.8	0.319
Baseline RBC, ${\mathrm{10}}^{12}$/L	4.6 ± 0.5	4.4 ± 0.5	4.6 ± 0.5	0.021
Post operation RBC, ${\mathrm{10}}^{12}$/L	4.4 ± 0.5	3.9 ± 0.6	4.4 ± 0.5	0.000
Baseline Hb, g/L	140.5 ± 14.8	135.8 ± 14.4	140.8 ± 14.8	0.030
Post operation Hb, g/L	132.4 ± 16.1	120.0 ± 17.6	133.2 ± 15.7	0.000
ΔHb, g/L	8.0 ± 8.7	15.6 ± 8.6	7.6 ± 8.5	0.000
PLT, 109/L	217.9 ± 62.7	217.0 ± 48.2	217.9 ± 63.3	0.736
Creatinine, mg/mL	0.83 ± 0.2	0.81 ± 0.2	0.84 ± 0.2	0.646
TC, mmol/L	4.5 ± 15.4	10.4 ± 43.4	4.3 ± 12.5	0.485
TG, mmol/L	1.8 ± 1.3	1.5 ± 0.6	1.8 ± 1.3	0.050
HDL-C, mmol/L	1.0 ± 0.2	0.9 ± 0.9	1.0 ± 0.2	0.357
LDL-C, mmol/L	2.2 ± 0.9	2.0 ± 1.0	2.2 ± 0.9	0.040
Hs-CRP, mg/L	3.4 ± 11.0	1.8 ± 3.3	3.5 ± 11.3	0.168
D-dimer, ng/mL	129.0 ± 204.6	120.9 ± 94.0	129.4 ± 208.9	0.294
BNP, pg/mL	100.8 ± 172.3	86.6 ± 110.2	101.6 ± 175.1	0.472

WBC, white blood cell; RBC, red blood cell; Hb, hemoglobin; PLT, platelet; TC, total cholesterol; TG, triacylglycerol; HDL-C, high-density lipoprotein cholesterol; LDL-C, low-density lipoprotein cholesterol; Hs-CRP, hypersensitive C-reactive protein; BNP, B-type natriuretic peptide.

### 3.3 Angiographic and Procedural Characteristics

Patient angiographic and procedural characteristics are summarized in Table 4. The average operative duration was higher among patients that experienced shock relative to patients that did not (144.4 ± 62.2 vs. 88.6 ± 48.8, *p *< 0.001). With respect to CTO target vessels, patients in the shock group exhibited lower LCX ratios (1 [1.4%] vs. 142 [16.1%], *p* = 0.013), while no differences were observed for other vessels. Patients in the shock group exhibited more complex anatomical features on CTO angiography, and presented with higher rates of distal cap at bifurcation (19 [43.2%] vs. 222 [25.2%], *p* = 0.008), proximal segment target (23 [52.3%] vs. 320 [36.4%], *p* = 0.033), CTO length ≥20 mm (38 [86.4%] vs. 610 [69.4%], *p* = 0.016), prior attempt (11 [25.0%] vs. 68 [7.8%], *p* = 0.001, and J-CTO score (2.5 ± 1.0 vs. 1.9 ± 1.1, *p *< 0.001). With respect to the CTO PCI operative procedures employed, femoral access (30 [68.2%] vs. 417 [47.4%], *p* = 0.007), a retrograde approach (25 [56.8%] vs. 111 [12.6%], *p *< 0.001), the knuckle technique (4 [9.4%] vs. 25 [2.8%], *p* = 0.044), and the externalization technique (16 [36.4%] vs. 53 [6.0%], *p *< 0.001) were more likely to be employed in the shock group relative to among patients that did not experience shock.

**Table 4.** **Angiographic and procedural characteristics**.

Variable	Overall	Shock		*p* value
	(n = 924)	Yes (n = 44)	No (n = 880)	
Procedure time, minute	91.2 ± 50.9	144.4 ± 62.2	88.6 ± 48.8	0.000
**Coronary artery dominance**				
Left dominant	56 (6.1%)	1 (2.3%)	55 (6.3%)	0.512
Right dominant	753 (81.5%)	39 (88.6%)	714 (81.1%)	0.211
Codominant	115 (12.4%)	4 (9.4%)	111 (12.6%)	0.490
Multivessel lesions	702 (76.0%)	34 (77.3%)	668 (75.9%)	0.836
Multiple CTO lesions	59 (6.4%)	1 (2.3%)	58 (6.6%)	0.355
ISR CTO	86 (9.3%)	6 (13.6%)	80 (9.1%)	0.289
**Target vessel**				
LAD	374 (40.5%)	18 (40.9%)	356 (40.5%)	0.952
LCX	143 (15.1%)	1 (1.4%)	142 (16.1%)	0.013
RCA	392 (42.4%)	25 (56.8%)	367 (41.7%)	0.048
Side branch at proximal cap	448 (48.5%)	24 (54.5%)	424 (48.2%)	0.410
Distal cap at bifurcation	241 (26.1%)	19 (43.2%)	222 (25.2%)	0.008
Proximal segment target	343 (37.1%)	23 (52.3%)	320 (36.4%)	0.033
Blunt/no stump	666 (72.1%)	37 (84.1%)	624 (70.7%)	0.069
Moderate/severe tortuosity	291 (31.5%)	19 (43.2%)	272 (30.9%)	0.087
CTO Length ≥20 mm	648 (70.1%)	38 (86.4%)	610 (69.4%)	0.016
Moderate/severe calcification	72 (7.8%)	4 (9.4%)	68 (7.8%)	0.770
Prior CTO PCI attempt	79 (8.5%)	11 (25.0%)	68 (7.8%)	0.001
No interventional collaterals	48 (5.2%)	0 (0%)	48 (5.5%)	0.162
Bad distal landing zone	30 (3.2%)	1 (1.4%)	29 (3.2%)	1.000
J-CTO score	1.9 ± 1.1	2.5 ± 1.0	1.9 ± 1.1	0.000
J-CTO score ≥2	631 (68.3%)	36 (81.8%)	595 (67.6%)	0.048
Use of femoral access	447 (48.4%)	30 (68.2%)	417 (47.4%)	0.007
Retrograde approach	136 (14.7%)	25 (56.8%)	111 (12.6%)	0.000
Knuckle technique	29 (3.1%)	4 (9.4%)	25 (2.8%)	0.044
Externalization technique	69 (7.5%)	16 (36.4%)	53 (6.0%)	0.000
Reverse-CART technique	16 (1.7%)	1 (1.4%)	15 (1.7%)	0.545
ADR(Stingray) technique	11 (1.2%)	1 (1.4%)	10 (1.1%)	0.417
Procedural success	789 (85.4%)	39 (88.6%)	750 (85.2%)	0.532

CTO, chronic total occlusion; ISR, in-stent restenosis; LAD, left anterior descending artery; LCX, left circumflex artery; RCA, right coronary artery; PCI, percutaneous coronary intervention; ADR, anterograde dissection reentry.

### 3.4 Risk Scoring Model Development

A multivariate stepwise logistic regression analysis identified six independent predictors of unexplained shock (Table 5), including baseline SBP (OR 0.968, 95% CI: 0.945–0.991), baseline heart rate (OR 1.055, 95% CI: 1.020–1.091), baseline Hb levels (OR 0.970, 95% CI: 0.947–0.994), operative duration (OR 1.008, 95% CI: 1.002–1.015), J-CTO score (OR 1.521, 95% CI: 1.021–2.267), and use of retrograde approach (OR 3.252, 95% CI: 1.426–7.415). Correspondnig adjusted odds ratios are shown in Table 6.

**Table 5.** **Multivariable analysis of patients in this study**.

Variable	Univariable analysis			Stepwise logistic regression		
	Shock	No shock	*p* value	Odds ratio	95% CI	*p* value
	(n = 44)	(n = 880)				
BMI, kg/$m^{2}$	25.3 ± 4.6	26.5 ± 3.5	0.049			
Baseline systolic pressure, mmHg	122.8 ± 19.0	128.1 ± 16.0	0.029	0.968	0.945–0.991	0.007
Baseline heart rate, /min	76.1 ± 12.5	71.7 ± 10.6	0.022	1.055	1.020–1.091	0.002
Baseline Hb, g/L	135.8 ± 14.4	140.8 ± 14.8	0.030	0.970	0.947–0.994	0.015
LDL-C, mmol/L	2.0 ± 1.0	2.2 ± 0.9	0.040			
Distal cap at bifurcation	19 (43.2%)	222 (25.2%)	0.008			
Proximal segment target	23 (52.3%)	320 (36.4%)	0.033			
Prior PCI	28 (63.6%)	424 (48.2%)	0.045			
Valvular regurgitation (moderate-severe)	5 (11.4%)	37 (4.2%)	0.044			
Procedure time, minute	144.4 ± 62.2	88.6 ± 48.8	0.000	1.008	1.002–1.015	0.008
J-CTO score	2.5 ± 1.0	1.9 ± 1.1	0.000	1.521	1.021–2.267	0.039
Use of femoral access	30 (68.2%)	417 (47.4%)	0.007			
Knuckle technique	4 (9.4%)	25 (2.8%)	0.044			
Retrograde approach	25 (56.8%)	111 (12.6%)	0.000	3.252	1.426–7.415	0.005
LCX target	1 (1.4%)	142 (16.1%)	0.013			
RCA target	25 (56.8%)	367 (41.7%)	0.048			

BMI, body mass index; LDL-C, low-density lipoprotein cholesterol; RBC, red blood cell; Hb, hemoglobin; LCX, left circumflex artery; RCA, right coronary artery; PCI, percutaneous coronary intervention.

**Table 6.** **Adjusted odds ratios for predictors of shock**.

Variable	Odds Ratio	95% CI	*p* value
Baseline systolic pressure (per + 10 mmHg)	0.721	0.568–0.915	0.007
Baseline heart rate (per + 10 /min)	1.704	1.219–2.381	0.002
Baseline Hb (per + 10 g/L)	0.741	0.583–0.943	0.015
Procedure time (per + 30 mins)	1.286	1.069–1.548	0.008
J-CTO score (per + 1 point)	1.521	1.021–2.267	0.039
Use of retrograde approach	3.252	1.426–7.415	0.005

Hb, hemoglobin.

**Fig. 2.**
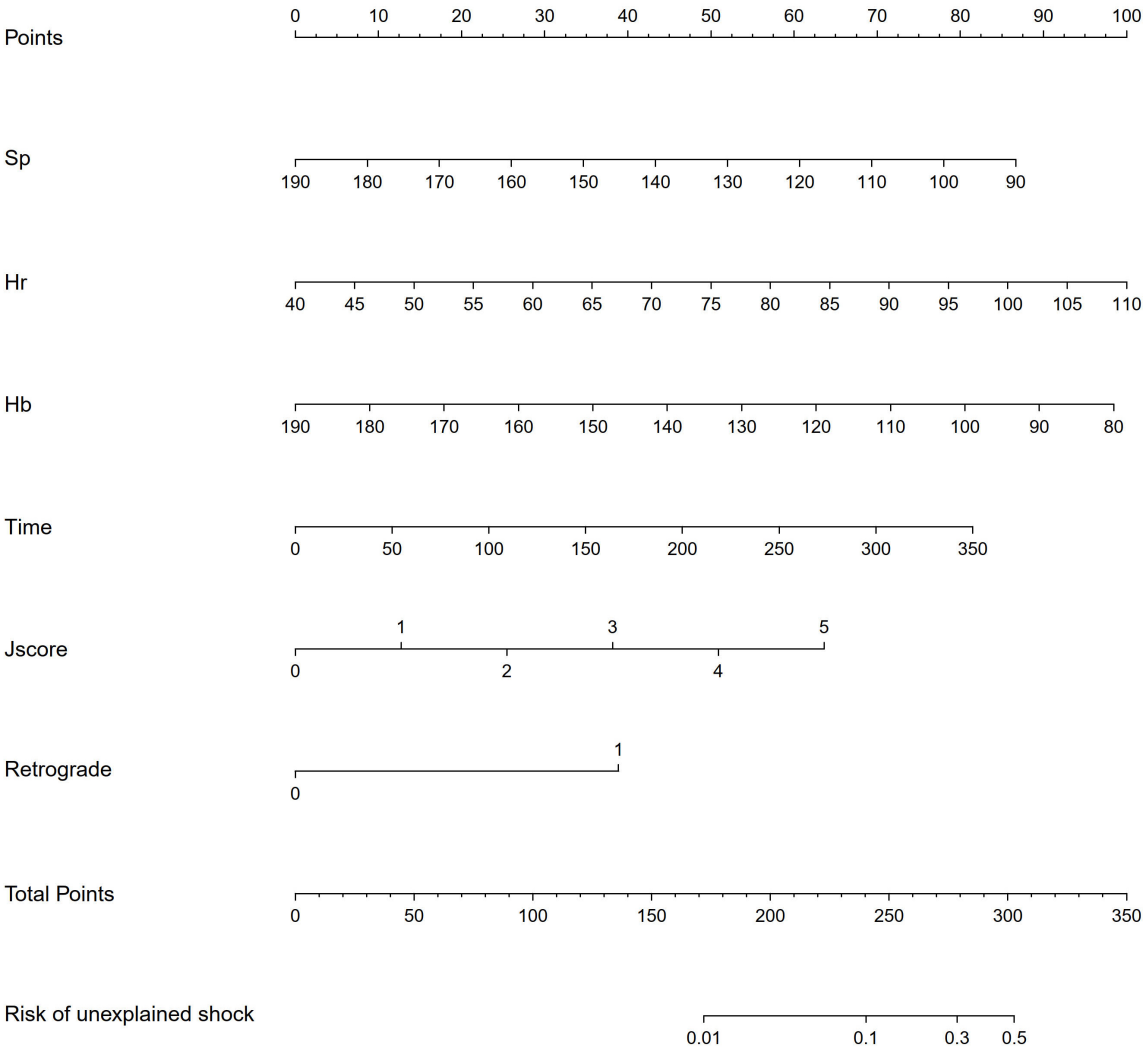
**Nomogram predicting the risk of unexplained shock based on dependent factors identified from multivariate logistic regression**. Sp, systolic pressure, mmHg (baseline); Hr, heart rate, /min (baseline); HB, hemoglobin, g/L (baseline); Time, Procedure time, minute.

**Fig. 3.**
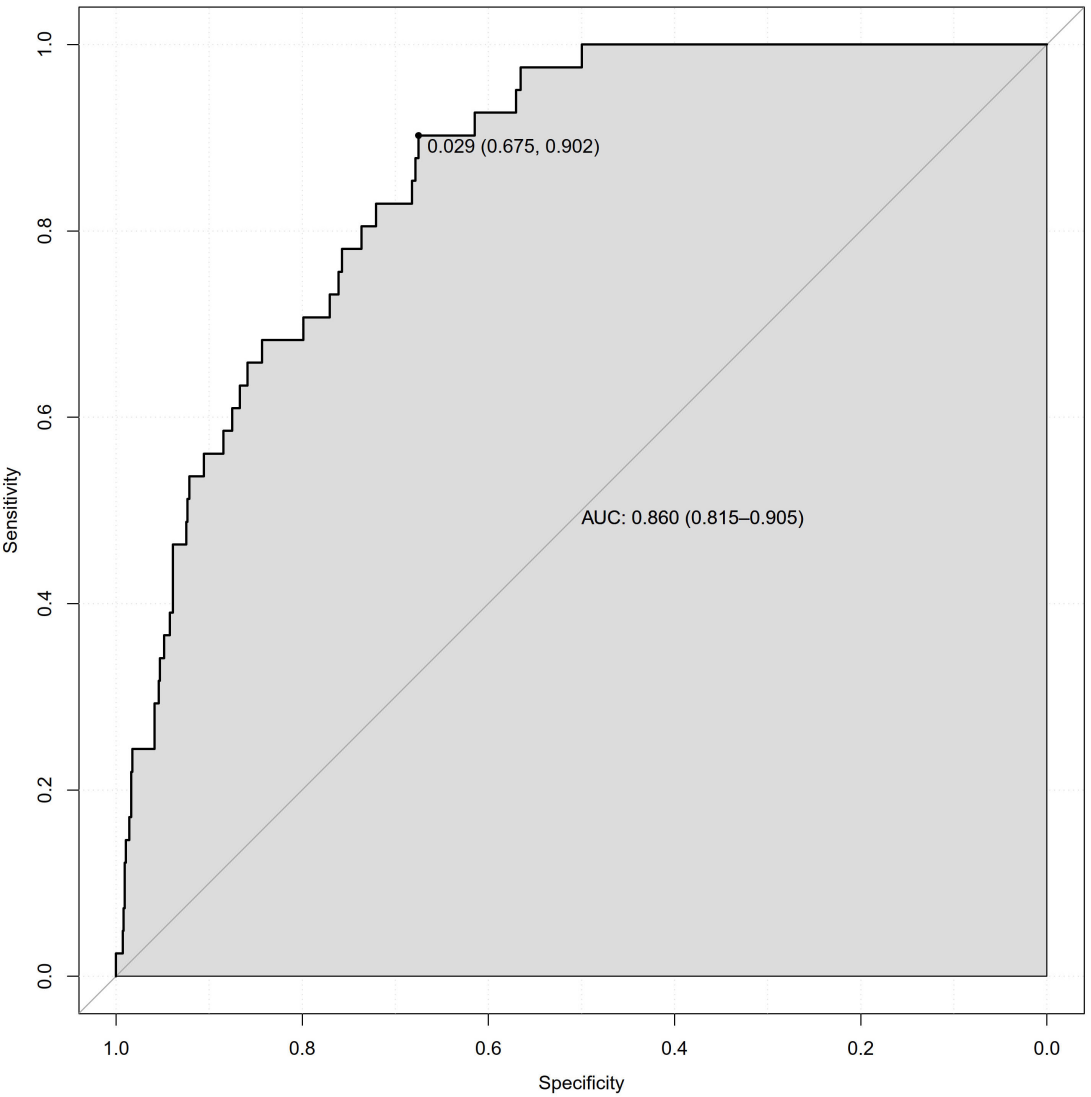
**ROC curve based on predicted probabilities obtained from the model**. AUC, area-under-the-curve; ROC, receiver operating characteristic.

These perioperative predictors were then used to design a nomogram capable of quantifying a given individual’s risk of experiencing unexplained shock (Fig. 2). ROC curves for the final model are shown in Fig. 3, with discrimination having been assessed based on an unbiased C-index estimate of 0.859.

## 4. Discussion

Unexplained shock is a potential CTO PCI procedural complication that has been the focus of insufficient study to date. The results of this study indicate that unexplained shock affected ~4.8% of CTO PCI patients, in addition to revealing 6 simple clinical indicators can be used to predict unexplained shock risk. This study is the first to our knowledge to have developed a model for the prediction of unexplained shock during CTO PCI, and these findings may be invaluable for future procedural planning efforts.

In a prior meta-analysis, a 3.1% pooled complication rate was reported for 18,061 cases [2], while a 2.8% complication rate was reported for 1569 hybrid CTO procedures in the PROGRESS registry [11]. The unexplained shock incidence rate for the 924 cases in this study (4.8%) was in line with the CTO complication rates reported previously [2]. This emphasizes the need for CTO operators to take this complication into consideration, given that shock-related data have not been reported for large CTO-related clinical studies such as the OPEN CTO study [12], EXPLORE study [13], and the EuroCTO study [14].

Shock is a clinical state wherein patients exhibit circulatory failure resulting in insufficient cellular oxygen utilization [15]. Shock is diagnosed based on a combination of hemodynamic, biochemical, and clinical findings [15]. Hypotension, which is common in the context of CTO PCI and can arise in response to many different factors, precedes shock [16, 17]. Certain causes of hypotension such as allergic reactions, vasovagal syndrome, guide interference with the aortic valve, or deep guide engagement can be alleviated through basic investigation and appropriate intervention [17]. Severe shock, however, is often caused by complications, and the differential diagnosis for complication-related shock is complex. Hypovolemic shock can arise due to access site complications and bleeding, while coronary complications such as donor vessel injury or perforation can exhibit a sudden and severe onset. When complication-related hypotension develops, it is vital that the underlying complications be rapidly treated to prevent progression to shock [18]. Most such complications are the result of the use of particular intraoperative procedures and techniques. Many different factors can contribute to the incidence of complications, and PROGRESS CTO complications scores offer value in the prediction of CTO PCI procedure-related complications [11]. Many patients suffering from shock, however, do not exhibit any apparent serious complications, with these cases being designated as instances of unexplained shock. Few studies to date have mentioned unexplained or complication-related shock when discussing CTO PCI-related procedural outcomes. As these two forms of shock may be driven by distinct underlying mechanisms, further efforts to differentiate between the two are warranted.

When comparing the two patient groups in this study, significant differences in base-line SBP, heart rate, BMI, and prior PCI were observed. The association between BP control and long-term PCI patient prognosis remains a matter of controversy [19]. However, prior evidence has revealed a link between lower BP at admission and in-hospital prognosis. Shiraish *et al*. [20] studied a population of Japanese acute MI patients undergoing PCI, and found an SBP <105 mmHg to be linked to a higher risk of in-hospital PCI patient mortality. Analyses of the INVEST study [21] supported a potential J-shaped relationship between SBP and MACE rates, while both SBP <125 mmHg on admission and heart rate >90 bpm are important risk factors associated with the incidence of cardiogenic shock during post-STEMI hospitalization as per the ORBI risk score [22]. Here, decreased BP and high heart rate were both significantly related to the risk of unexplained shock. Several factors may explain these findings. For one, more rapid heart rate on admission is indicative of reduced cardiac reserve [23], such that patients may be less capable of compensating for blood loss or ischemia. In addition, decreased BP on admission may be linked to impaired cardiac function and hypovolemia [24]. While lower body weight has previously been linked to an increased risk of bleeding for individuals from Asian populations [25], BMI failed to offer value in the prediction of unexplained shock in the present study cohort. This may be attributable to the fact that most blood loss in these patients was CTO PCI procedure-related, rather than complication-induced.

As CTO PCI necessitates the utilization of large sheaths and is associated with a high frequency of dual access, it is associated with a high risk of blood loss [26]. Procedure-associated blood loss may represent an important cause of unexplained shock incidence. Here, all cases exhibited a mean decrease in hemoglobin levels of ~8 g/L (140.5 ± 14.8 to 132.4 ± 16.1), and these decreases were more pronounced in the shock group (140.8 ± 14.8 to 133.2 ± 15.7). Such procedure-related blood loss is in part attributable to more frequent instrument exchanges from the Y-connector, and strategies including retrograde wire externalization further exacerbate this risk [27].

The risk of ischemia is higher for the CTO PCI procedure as compared to conventional PCI [16], in part owing to the use of additional contrast agents and a guiding catheter with a larger diameter. Certain techniques including retrograde PCI can contribute to donor artery or collateral channel ischemia [17]. While these events may not result in serious complications, they do decrease cardiac output. The extent to which these is- chemic risks are impacted following CTO opening remains to be established.

Several studies have identified severe cardiac insufficiency or low LV function (LVEF ≤30%) as important criteria for high-risk PCI [28]. However, all patients in the present study exhibited an ejection fraction of >40%, potentially owing to the patient selection criteria and adequate OMT therapy employed herein.

J-CTO scores are correlated with CTO complexity, with CTOs exhibiting a J-CTO score ≥2 being associated with a higher risk of necessitating more complex antegrade techniques and retrograde crossing techniques [29]. Many studies have reported the retrograde approach to be predictive of procedure-related complications [6, 11, 30, 31]. While this retrograde technique is often necessary to ensure high rates of technical success, it is highly complex and associated with the potential for complications including donor vessel ischemia, donor vessel injury, or collateral injury. Moreover, the retrograde approach necessitates a longer target activated clotting time (ACT, >350 s), elevating the potential risk of bleeding. Minimizing the procedure duration when possible is a core tenant of the CTO PCI approach [32], but these complex techniques inevitably prolong the operation time, thereby extending the duration of bleeding and ischemia.

Overall, these findings suggest that a combination of ischemia and blood loss due to a variety of reasons can contribute to the incidence of unexplained shock among patients undergoing the CTO PCI procedure. While these patients may not experience complications in the traditional sense that are directly related to procedural success, efforts to mitigate CTO PCI-related blood loss and ischemia may protect against the incidence of unexplained shock. The risk scoring system developed herein has the potential to aid clinicians performing the CTO PCI procedure by enabling appropriate preoperative planning, arrangement, and strategy adjustment as necessary. The risk of shock for high-risk patients can be mitigated by reducing the operative duration to the greatest extent possible, employing retrograde techniques, and allowing more skilled operators to perform the procedure.

## 5. Limitations

There are some limitations to this analysis. For one, this was a single-center retrospective study, and the results may thus not be representative of findings for other centers or operators. In addition, the mechanisms underlying unexplained shock were not clarified through this study, and further efforts to delineate these mechanisms may be critical to the treatment or prevention of this potentially serious clinical outcome. Moreover, these results do not offer any insight regarding long-term patient prognosis. Accordingly, we plan to perform future studies examining the mechanisms governing the incidence of unexplained shock and the long-term prognosis of these patients.

## 6. Conclusions

In summary, these results suggest that baseline systolic pressure, baseline heart rate, baseline hemoglobin levels, operative duration, J-CTO score, and the use of a retrograde approach can be used to predict the incidence of non-complication-related shock in patients undergoing CTO PCI procedures. These findings can be used to facilitate the preoperative evaluation of high-risk patients and corresponding strategy adjustment efforts.
